# *Interleukin-28B* Polymorphisms Predict the Efficacy of Peginterferon Alpha in Patients With Chronic Hepatitis B: A Meta-Analysis

**DOI:** 10.3389/fmed.2021.691365

**Published:** 2021-07-09

**Authors:** Sang-Yu Ying, Yao-Ren Hu, Guo-Sheng Gao, Ke-Hong Lou, Zhen Huang

**Affiliations:** ^1^Clinical Laboratory, HwaMei Hospital, University of Chinese Academy of Sciences, Ningbo, China; ^2^Ningbo Institute of Life and Health Industry, University of Chinese Academy of Sciences, Ningbo, China; ^3^Department of Hepatology, HwaMei Hospital, University of Chinese Academy of Sciences, Ningbo, China; ^4^Department of Blood Transfusion, HwaMei Hospital, University of Chinese Academy of Sciences, Ningbo, China

**Keywords:** IL-28B, polymorphism, hepatitis B virus, polyethylene glycol, interferon alpha, meta-analysis

## Abstract

**Background:** Polyethylene glycol interferon alpha (PEG-IFN-α) is the most frequently used pharmacotherapeutic approach in patients infected with hepatitis B virus (HBV). Numerous studies have reported that *interleukin-28B (IL-28B)* genetic polymorphisms are related to the therapeutic efficacy of PEG-IFN-α, but the results are inconsistent. The present meta-analysis aimed to analyze the association between *IL-28B* genetic polymorphisms and the prognosis of patients with chronic hepatitis B (CHB) treated with PEG-IFN-α to inform clinical practice.

**Methods:** PubMed, EBSCO, and Scopus databases were searched for relevant literature published before February 30, 2021. We calculated the crude odds ratios (ORs) with 95% confidence intervals (CIs) of the cited articles. A total of 2510 patients with CHB treated with PEG-IFN-α in 13 clinical cohort studies were analyzed.

**Results:** The overall analysis demonstrated a potential association between *IL-28B* genetic polymorphisms and response to PEG-IFN-α; however, the association was not statistically significant. Furthermore, the subgroup analysis revealed that among patients with HBeAg-negative CHB, the *rs12979860* CC genotype and *rs8099917* TT genotype were associated with more significant treatment response to PEG-IFN-α (CC vs. non-CC: OR 2.78, 95% CI 1.00–7.76, *I*^2^ = 83%; TT vs. non-TT: OR 2.16, 95% CI 1.35–3.48, *I*^2^ = 0%). Among Asian patients with CHB, the *rs12979860* CC genotype was associated with a more significant treatment response to PEG-IFN (CC vs. non-CC: OR 1.88, 95% CI 1.18–2.99, *I*^2^ = 0%).

**Conclusion:** This meta-analysis revealed that the *IL-28B rs12979860* CC genotype and *rs8099917* TT genotype indicated a better treatment response than non-CC and non-TT genotypes for PEG-IFN-α in patients with CHB.

## Introduction

Hepatitis B vaccine programs have been implemented gradually. Hepatitis B virus (HBV) infection affects more than 350 million people worldwide and remains the most common cause of liver cancer and has a very low five-year survival rate (1, 2). More than 2000 million patients infected with HBV die every year due to HBV-related diseases, such as hepatocellular carcinoma (HCC) and liver cirrhosis (1). Currently, the prevention of HBV injection remains dominant in the treatment of chronic hepatitis B (CHB) and other HBV-related diseases. However, HBV complete eradication is difficult to achieve once a person is infected by HBV without effective prevention. Thus, the current therapeutic strategy for patients infected with HBV is to prevent CHB-related complications, which can be achieved by suppressing HBV replication. PEG-IFN-α is a current therapeutic strategy for patients with CHB with a remarkable effect in HBeAg-negative adults (3–5). Presently, treatment with IFN for more than 1 year is the primary therapy for adult patients with CHB, achieving complete HBsAg clearance (6). Furthermore, IFN-associated therapies also achieve a sustainable therapeutic response in most patients with CHB after 48-weeks of treatment (7, 8). However, previous studies found that the therapeutic results of PEG-IFN-α treatment in patients with CHB with HBeAg seroconversion or loss are poor (9, 10). Thus, it is essential to select patients with CHB who are sensitive to PEG-IFN-α so that PEG-IFN-α-based treatment achieves a better curative effect.

It is well-known that cytokines and regulatory molecules play critical roles in the immune response and pathogenesis of HBV infection. Interferon lambda 3 (IFNL3) is a cytokine encoded by *interleukin 28B* (*IL-28B*) and exerts anti-viral effects on HBV replication (11, 12). Based on the anti-viral and immune actions of *IL-28B*, researchers predicted that *IL-28B* genotypes might be associated with HBV infection and the therapeutic efficacy of interferons in patients with CHB. However, another study found three single-nucleotide polymorphisms (SNPs), *rs12979860* C/T, *rs12980275* A/G, and *rs8099917* T/G, of *IL-28B* are not associated with the outcomes of HBV infection. Furthermore, Zhao et al. performed a meta-analysis to evaluate the role of *IL-28B* SNPs (*rs12979860, rs12980275*, and *rs8099917*) in the progression of HBV infection and the results showed that *IL28B* polymorphisms had no association with the outcome of HBV infection (13). However, the association between IL28B polymorphisms and the efficacy of PEG-IFN-α in patients with CHB was not analyzed. It is encouraging that Wu et al. found that *IL-28B* polymorphisms could predict the clinical outcomes of PEG-IFN-α in Chinese patients with CHB (14). However, another study performed in Asian populations found that the *IL-28B* genotype is not accurate in predicting outcomes in patients infected with HBVs treated with PEG-IFN-α (15). Thus, it is imperative to further analyze the association between *IL-28B* polymorphisms and the prognosis of patients with CHB treated with PEG-IFN-α. We then designed and performed this meta-analysis to elaborate the association between *IL-28B* genetic polymorphisms and the prognosis of patients with CHB treated with PEG-IFN-α to inform clinical practice.

## Methods

### Search Strategy and Selection Criteria

This meta-analysis was conducted according to the Preferred Reporting Items for Systematic Reviews and Meta-Analyses (PRISMA) guidelines (15). Two authors independently searched PubMed, Embase, and Scopus until February 30, 2021, for relevant articles using the following keywords: peginterferon alpha, *IL-28B*, and hepatitis B. There were no language or data restrictions.

The inclusion criteria were as follows: (1) studies of patients with HBV infection; (2) patients receiving peginterferon alpha (PEG-IFN-α) therapy; (3) studies reporting precise *IL28B* genotypes (CC vs. CT + TT for *rs12979860*; AA vs. AG + GG for *rs12980275*; TT vs. GT + GG for *rs8099917*) for included patients; (4) the primary outcome was the treatment response, including virological response (HBV DNA < 2,000 IU/mL), serological response (HBeAg seroconversion), biochemical response (ALT or AST < 40 IU/L), or combined response; and (5) study design including randomized controlled trials, non-randomized controlled trials, observational study. In addition, case reports, non-human studies, studies without adequate information, or concerning outcomes were excluded from the meta-analysis.

### Data Extraction and Quality Assessment

Two authors independently retrieved and extracted relevant studies. The basic characteristics of the studies (first author, year of publication, ethnicity, sample size, population characteristics, genotypes, and definition of outcomes) were recorded in Table 1. Any discrepancies in all phases were resolved through a team consensus. If relevant information was not reported in the article, we contacted the corresponding authors for further information.

**Table 1:** Characteristics of included studies.

**References**	**Ethnicity**	**Sample size**	**Patients' characteristics**	**SNPs**	**Genotypes**	**Definition of outcome**	**NOS score**
Wei et al. (16)	Caucasian and Asian	701	CHB Patients treated with 48 weeks of PEG-IFN alfa-2a 180 μg/week in three RCTs	rs12979860, rs12980275, rs8099917	CC vs. non-CC, AA vs. non-AA, TT vs. non-TT	HBeAg seroconversion plus HBV DNA < 2,000 IU/ml in HBeAg-positive patients, and HBV DNA < 2,000 IU/ml in HBeAg-negative patients (24 weeks after end of treatment)	7
Domagalski et al. (17)	Caucasian	52	HBeAg-negative CHB children treated with PEG-IFN alfa-2a 180 μg/week for 48 weeks	rs12979860, rs12980275, rs8099917	CC vs. non-CC, AA vs. non-AA, TT vs. non-TT	HBV DNA level < 2,000 IU/mL and normalization of ALT activity < 40 IU/L at the 24 weeks post treatment	6
Limothai et al. (18)	Asian	107	HBeAg-positive CHB patients treated with PEG-IFN alfa-2a 180 μg/week for 48 weeks	rs12979860	CC vs. non-CC	HBeAg clearance plus HBV DNA < 2,000 IU/ml at 24 weeks post treatment	7
Boglione et al. (19)	Caucasian, Asian, African	190	HBeAg-negative CHB patients treated with PEG-IFN a-2a 180 μg/week for at least 48 weeks	rs12979860, rs12980275, rs8099917	CC vs. non-CC, AA vs. non-AA, TT vs. non-TT	HBV DNA < 2000 IU/mL at end of therapy	6
Domagalski et al. (20)	Caucasian	86	HBeAg-positive CHB patients, treated with PEG-IFN alfa-2a at a dose of 180 μg per week for 48 weeks	rs12979860, rs8099917	CC vs. non-CC, TT vs. non-TT	HBV DNA < 2,000 IU/mL at 24 weeks after treatment	7
Wu et al. (14)	Asian	212	HBeAg-positive CHB patients treated with PEG-IFN monotherapy	rs12979860, rs12980275, rs8099917	CC vs. non-CC, AA vs. non-AA, TT vs. non-TT	HBV DNA levels < 200 IU/ml and HBeAg seroconversion after 48 weeks after treatment	7
Zhang et al. (21)	Caucasian	97	HBeAg-positive CHB patients treated with PEG-IFN IFN	rs12979860	CC vs. non-CC	HBV DNA level < 2,000 IU/mL and HBeAg seroconversion 48 weeks after treatment discontinuation	7
Lampertico et al. (8)	Caucasian	101	HBeAg-negative CHB patients treated with PEG-IFN alfa-2a 180 μg/week	rs12979860	CC vs. non-CC	HBV DNA levels < 200 IU/ml 24 weeks after treatment	7
Guo et al. (22)	Asian	146	Patients with dual HBV/HCV infection who had PEG-IFN-α therapy 180 μg/week for 24 weeks	rs12979860, rs8099917	CC vs. non-CC, TT vs. non-TT	HBV DNA levels < 200 IU/ml 24 weeks post treatment	6
Holmes et al. (15)	Caucasian, Asian, African	96	Adult CHB patients treated with 48 weeks of PEG -IFN monotherapy	rs12979860	CC vs. non-CC	HBeAg seroconversion with HBV DNA < 2,000 IU/mL in HBeAg-positive patients or HBV DNA < 2,000 IU/mL in HBeAg-negative patients at 24 weeks post treatment	7
Wu et al. (23)	Asian	512	HBeAg-negative CHB patients received PEG-IFN-a-2a 180 μg/week for 12 months	rs8099917	TT vs. non-TT	ALT and AST levels < 40 IU/L, HBV DNA < 500 copies/mL, HBeAg seroconversion 24 weeks after therapy	6
de Niet et al. (24)	Caucasian, Asian, African	95	CHB patients treated with PEG-IFN alfa-2a 180 mg/week for 48 weeks	rs12979860	CC vs. non-CC	HBV DNA < 2,000 IU/mL and persistent normal ALT levels at 24 weeks after stopping therapy	6
Tseng et al. (25)	Asian	115	HBeAg-positive CHB patients treatment with PEG-IFN-a-2a 180 μg/week	rs8099917	TT vs. non-TT	HBeAg seroconversion, and HBV DNA < 2,000 IU/mL at 24 weeks post treatment	7

*RCT, randomized controlled trial; CHB, chronic hepatitis B; PEG-IFN, peginterferon; HBV, hepatitis B virus; DNA, deoxyribonucleic acid; IU, international unit; ALT, alanine aminotransferase; AST, aspartate aminotransferase; SNP, single nucleotide polymorphism; NOS, Newcastle-Ottawa scale*.

Two reviewers independently used the Newcastle-Ottawa Scale (NOS) to assess the risk of bias in the included studies. Publication bias was evaluated using Egger's regression test. Any discrepancies in all phases were resolved through a team consensus.

### Statistical Synthesis and Analysis

Pooled analysis was performed to calculate the odds ratio (OR) with a 95% confidence interval (95% CI) between genotypic variations in *IL-28B* and treatment response of patients with HBV infection receiving PEG-IFN-α therapy. We calculated the *I*^2^ statistic to measure the proportion of total variation in the study estimates attributed to heterogeneity. *I*^2^ values of < 25, 25–75, and >75% indicate low, moderate, and high heterogeneity, respectively (26). If significant heterogeneity existed, we adopted a random-effects model to perform the analysis. Subgroup analysis was performed according to HBeAg (HBeAg negative vs. positive) and race (Asian vs. Caucasian). All analyses were performed using RevMan 5.3 and R 3.6. Statistical significance was set at P < 0.05.

## Results

### Study Characteristics

**Figure 1:**
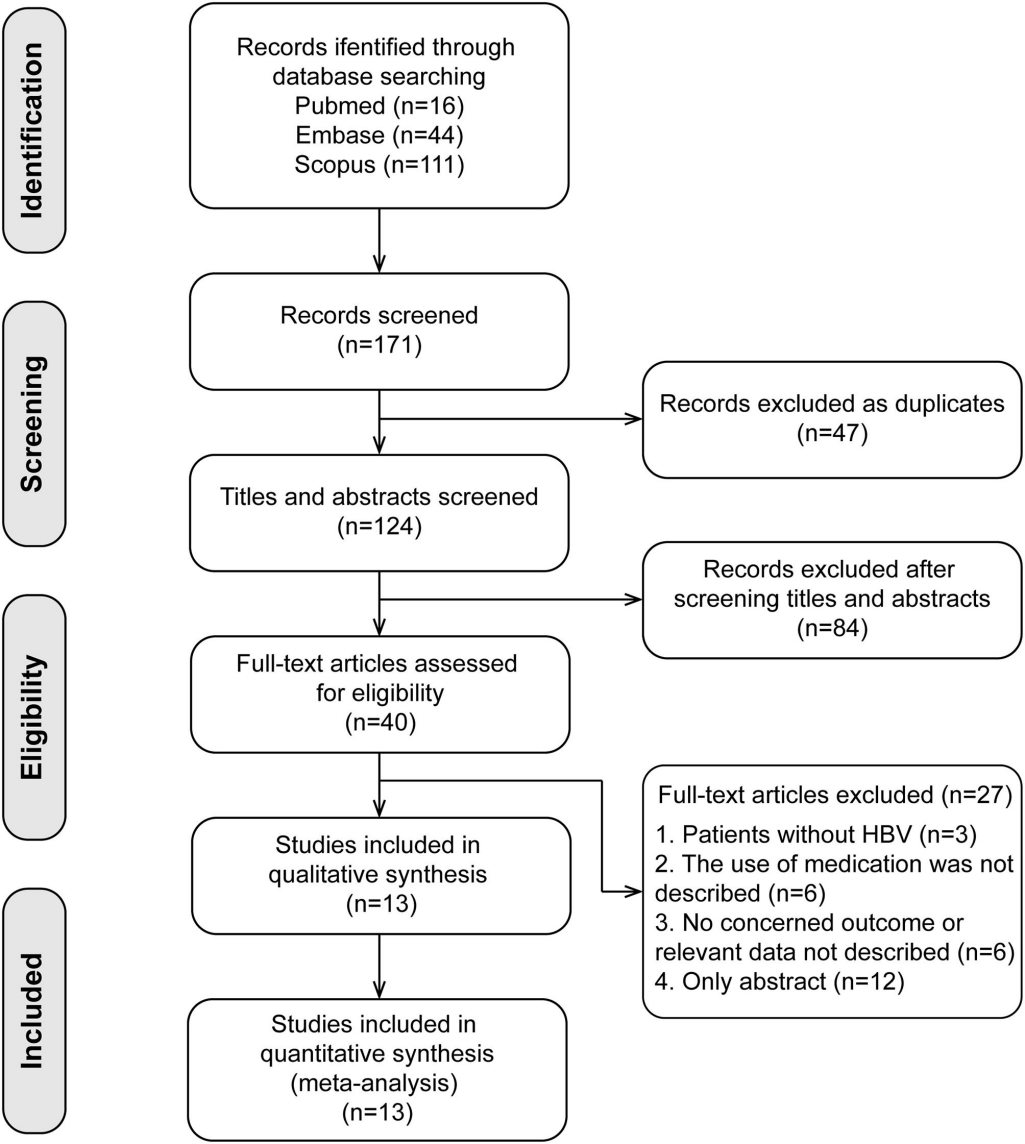
Flow chart of research selection process. Thirteen cohort studies were included in the present meta-analysis.

The search and selection processes are presented in Figure 1. A total of 171 studies were initially identified, and 47 duplicates were excluded. After screening the titles and abstracts, 40 full-text articles were assessed for eligibility. Finally, 13 studies involving 2,510 patients were included in the meta-analysis (14–25, 27). These included studies were published between 2011 and 2020, with sample sizes ranging from 52 to 701. There were four studies in the Caucasian populations, five in Asian populations, and four in admixture populations, including Caucasian, Asian, and African populations. All studies included patients with persistent HBV infection with PEG-IFN-α treatment, and three SNPs (*rs12979860, rs12980275*, and *rs8099917*) were genotyped using polymerase chain reaction. All studies reported the treatment response defined as HBV DNA < 2,000 IU/ mL for different genotype groups and nine studies combined the virological with serological or biochemical response as the primary outcome.

**Figure 2:**
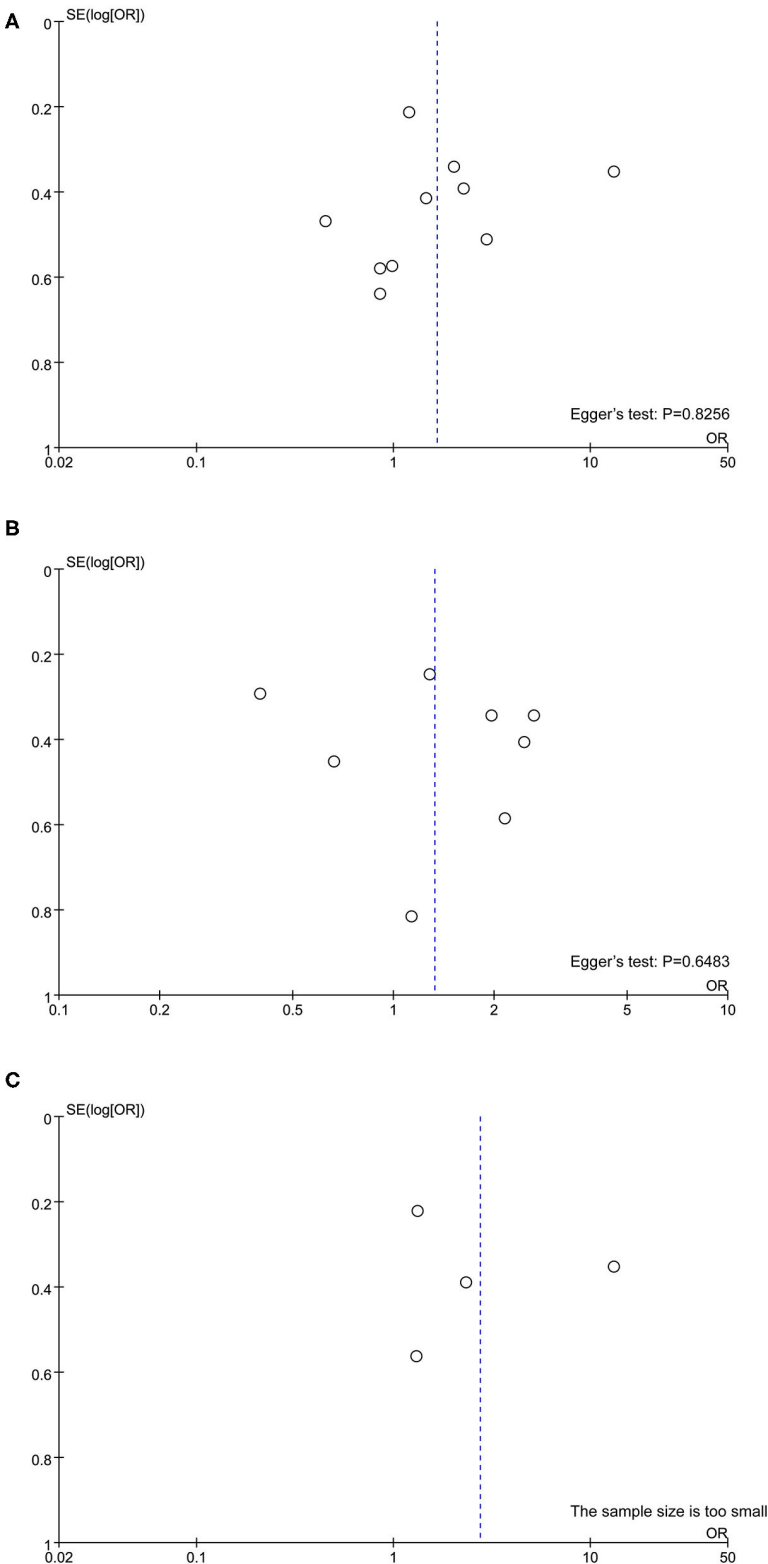
Publication bias assessed by funnel plot and Egger's test. **(A)** rs12979860; **(B)** rs8099917; **(C)** rs12980275.

Quality assessment using the NOS score is shown in Table 2. Ten studies (14–16, 18, 20, 21, 23–25, 27) were of high quality with a total score of >6. The comparability of cohorts is a major concern. Since none of the studies included in this meta-analysis were randomized trials, patients' characteristics such as age, sex, race, baseline HBV DNA, and medication history in different groups could not be fully controlled. In addition, Domagalski et al. (17) enrolled children with HBV, and Guo et al. (22) enrolled patients with dual HBV and HCV infection. The follow-up period in the trial of Boglione et al. (19) was not long enough for the outcome to occur. Visual inspection of funnel plots and Egger's test showed no significant publication bias (Figure 2).

**Table 2:** The quality assessment of included studies by the Newcastle-Ottawa Scale.

**Study**	**Newcastle-Ottawa scale components**								**Quality score**
	**1**	**2**	**3**	**4**	**5**	**6**	**7**	**8**	
Wei et al. (16)	^*^	^*^	^*^	^*^		^*^	^*^	^*^	7
Domagalski et al. (17)		^*^	^*^	^*^		^*^	^*^	^*^	6
Limothai et al. (18)	^*^	^*^	^*^	^*^		^*^	^*^	^*^	7
Boglione et al. (19)	^*^	^*^	^*^	^*^		^*^		^*^	6
Domagalski et al. (20)	^*^	^*^	^*^	^*^		^*^	^*^	^*^	7
Wu et al. (14)	^*^	^*^	^*^	^*^		^*^	^*^	^*^	7
Zhang et al. (21)	^*^	^*^	^*^	^*^		^*^	^*^	^*^	7
Lampertico et al. (27)	^*^	^*^	^*^	^*^		^*^	^*^	^*^	7
Guo et al. (22)		^*^	^*^	^*^		^*^	^*^	^*^	6
Holmes et al. (15)	^*^	^*^	^*^	^*^		^*^	^*^	^*^	7
Wu et al. (23)	^*^	^*^	^*^	^*^		^*^	^*^	^*^	7
de Niet et al. (24)	^*^	^*^	^*^	^*^		^*^	^*^	^*^	7
Tseng et al. (25)	^*^	^*^	^*^	^*^		^*^	^*^	^*^	7

*1, Representativeness of the exposed cohort; 2, Selection of the non-exposed cohort; 3, Ascertainment of exposure; 4, Demonstration that outcome of interest was not present at start of study; 5, Comparability of cohorts on the basis of the design or analysis; 6, Assessment of outcome; 7, Was follow-up long enough for outcomes to occur; 8, Adequacy of follow up of cohorts*.

* *p < 0.05*.

### Primary Analysis

**Figure 3:**
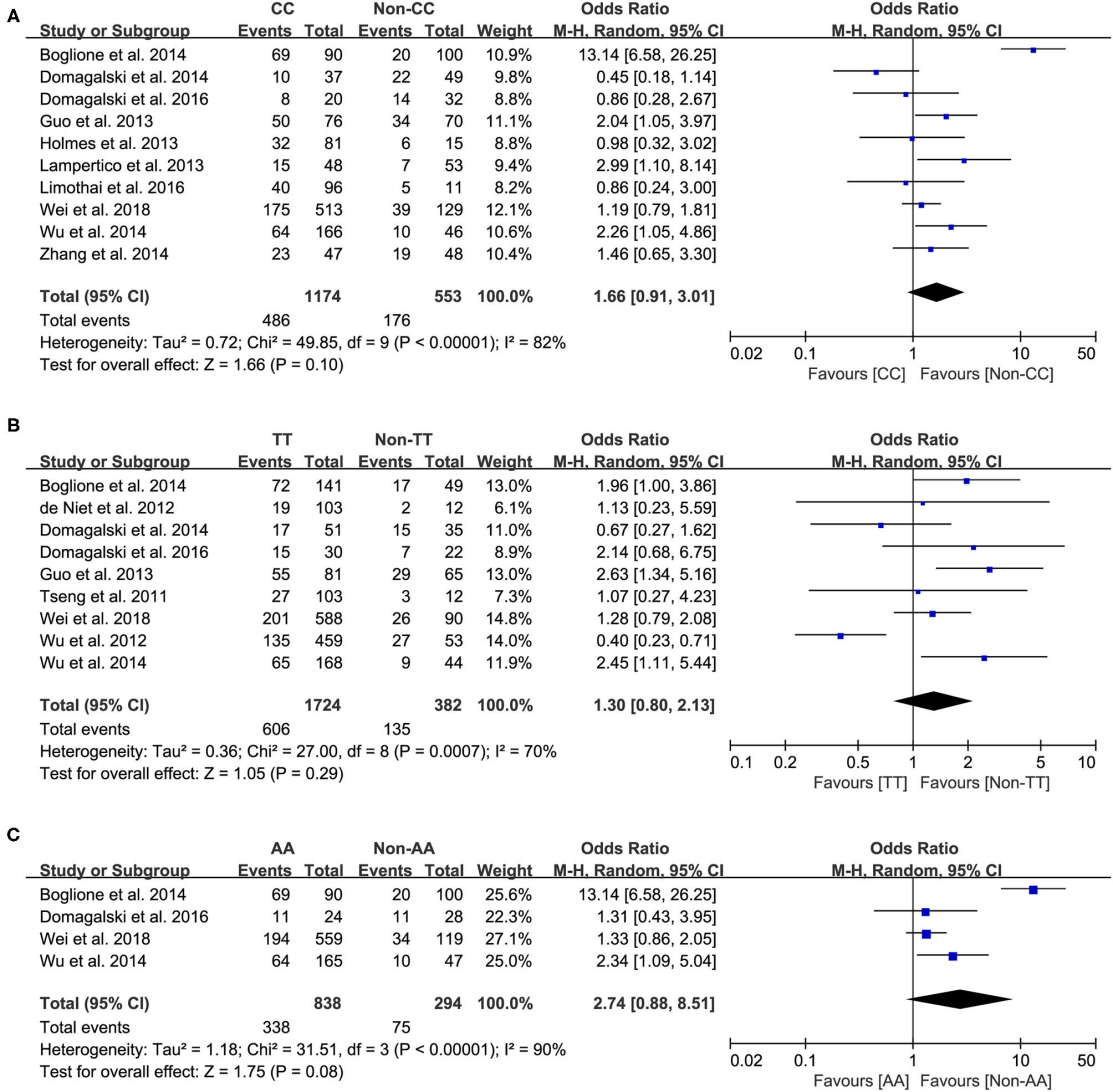
Forest plots for the association between IL-28B polymorphisms and treatment response of HBV infected patients treated with PEG-IFN. **(A)** overall analysis of rs12979860; **(B)** overall analysis of rs8099917; **(C)** overall analysis of rs12980275. HBV, hepatitis B virus; PEG-IFN, pegylated interferon.

Ten studies comprising 1,174 patients with CC genotype vs. 553 patients with non-CC genotype (including CT+TT) that considered *rs12979860*; 9 studies comprising 1724 patients with TT genotype vs. 382 patients with non-TT genotype (including TG+GG) for *rs8099917*; 4 studies comprising 838 patients with AA genotype vs. 294 patients with non-CC genotype (including AG+GG) for *rs12980275*. A random-effects model was employed to estimate the SNP polymorphism's association with the treatment response among patients with CHB with PEG-IFN-α treatment (Figure 3). There was no significant association between *rs12979860* and treatment response to PEG-IFN-α in all patients with HBV infection (CC vs. non-CC: OR 1.66, 95% CI 0.91–3.01, *I*^2^ = 82%). Similarly, no significant association was observed between *rs8099917* (TT vs. non-TT: OR 1.30, 95% CI 0.80–2.13, *I*^2^ = 70%) and *rs12980275* (AA vs. non-AA: OR 2.74, 95% CI 0.88–8.51, *I*^2^ = 90%).

### Subgroup Analysis

**Figure 4:**
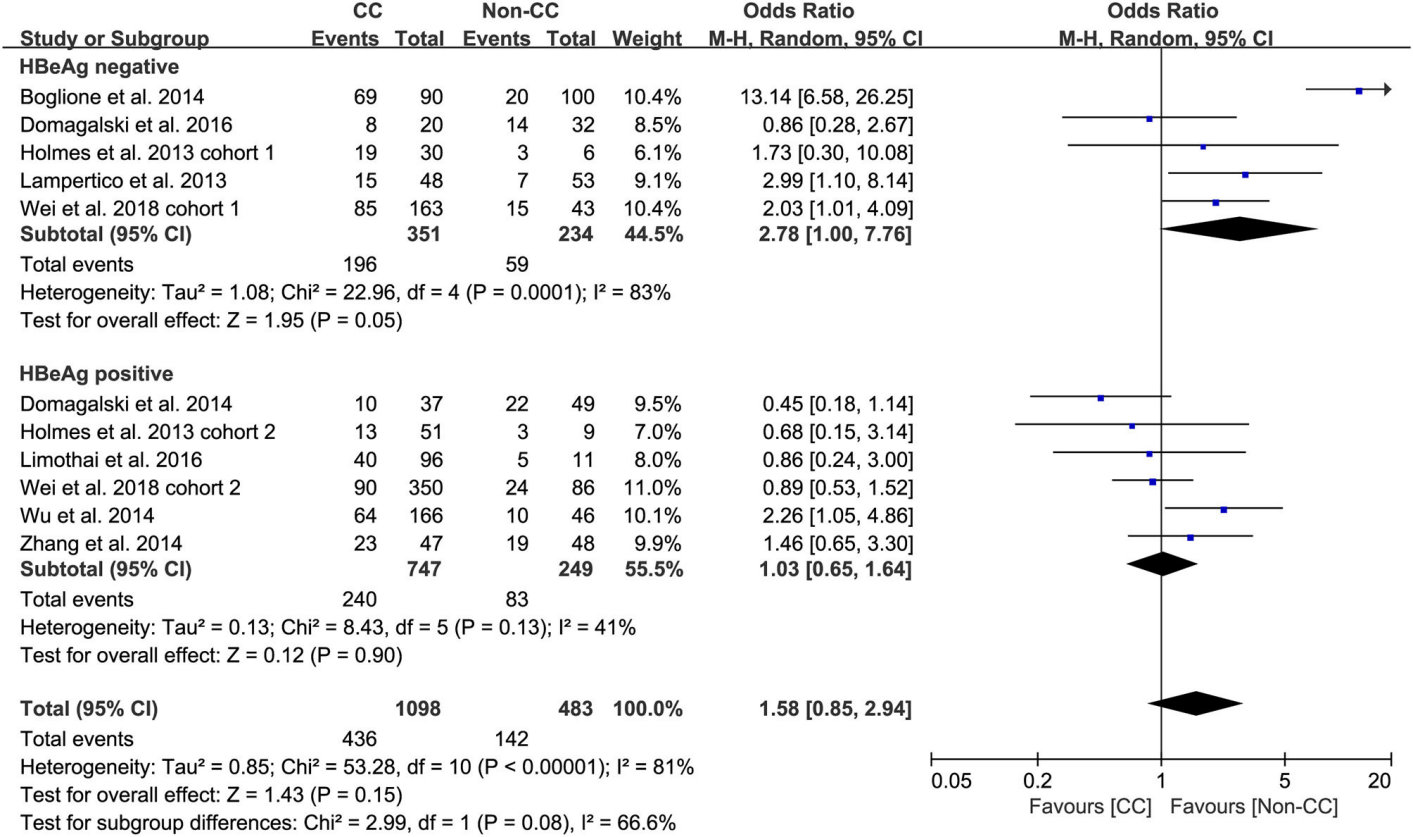
Subgroup analysis of rs12979860, stratified by HBeAg.

**Figure 5:**
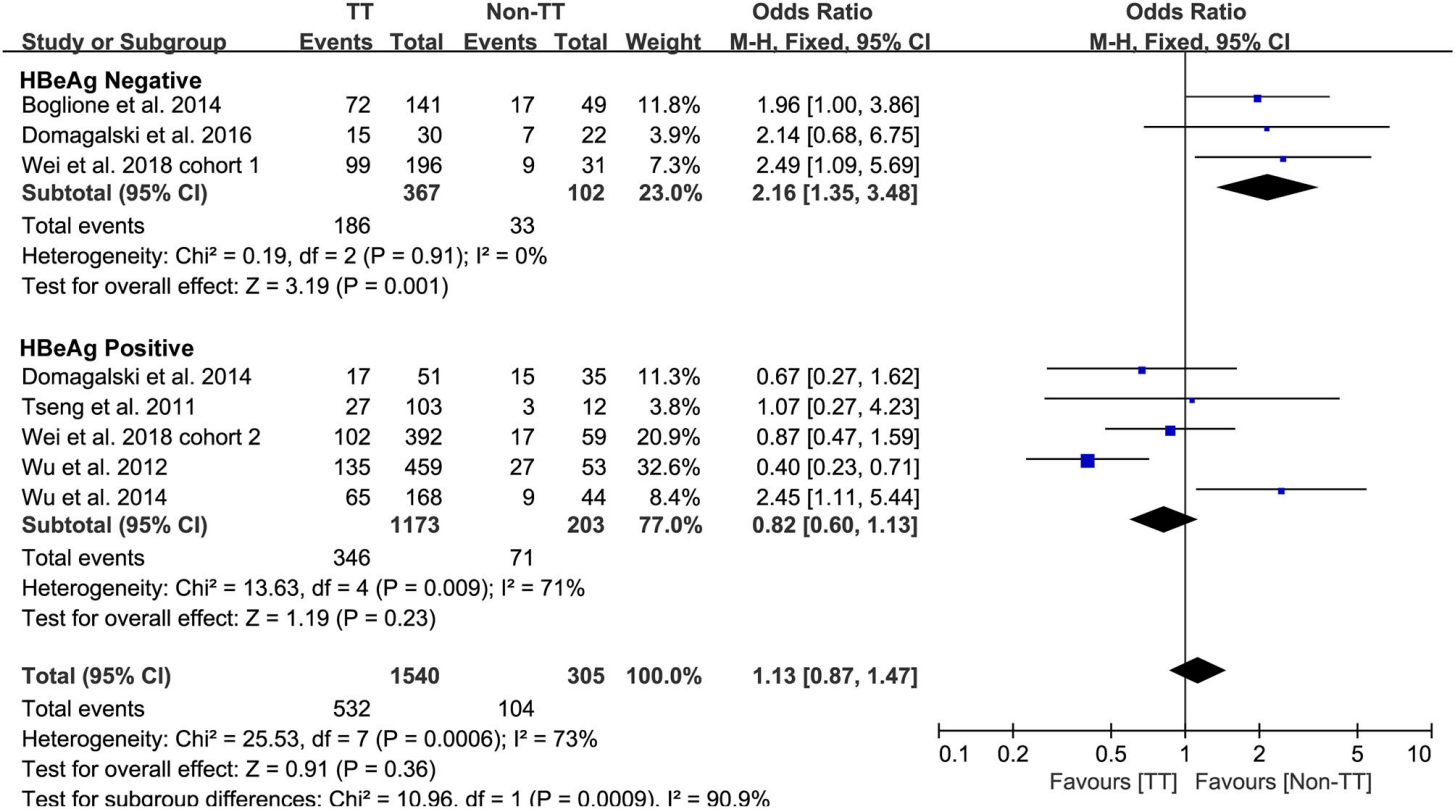
Subgroup analysis of rs12979860, stratified by HBeAg.

Predefined subgroup analyses were stratified by HBeAg and race to explore the discrepant treatment effect of different subgroups and heterogeneity. Among HBeAg-negative patients with CHB, the *rs12979860* CC genotype and *rs8099917* TT genotype were associated with a more significant treatment response to PEG-IFN-α (CC vs. non-CC: OR 2.78, 95% CI 1.00–7.76, *I*^2^ = 83%, Figure 4; TT vs. non-TT: OR 2.16, 95% CI 1.35–3.48, *I*^2^ = 0%, Figure 5). However, no differences were observed among patients with HBeAg-positive CHB.

**Figure 6:**
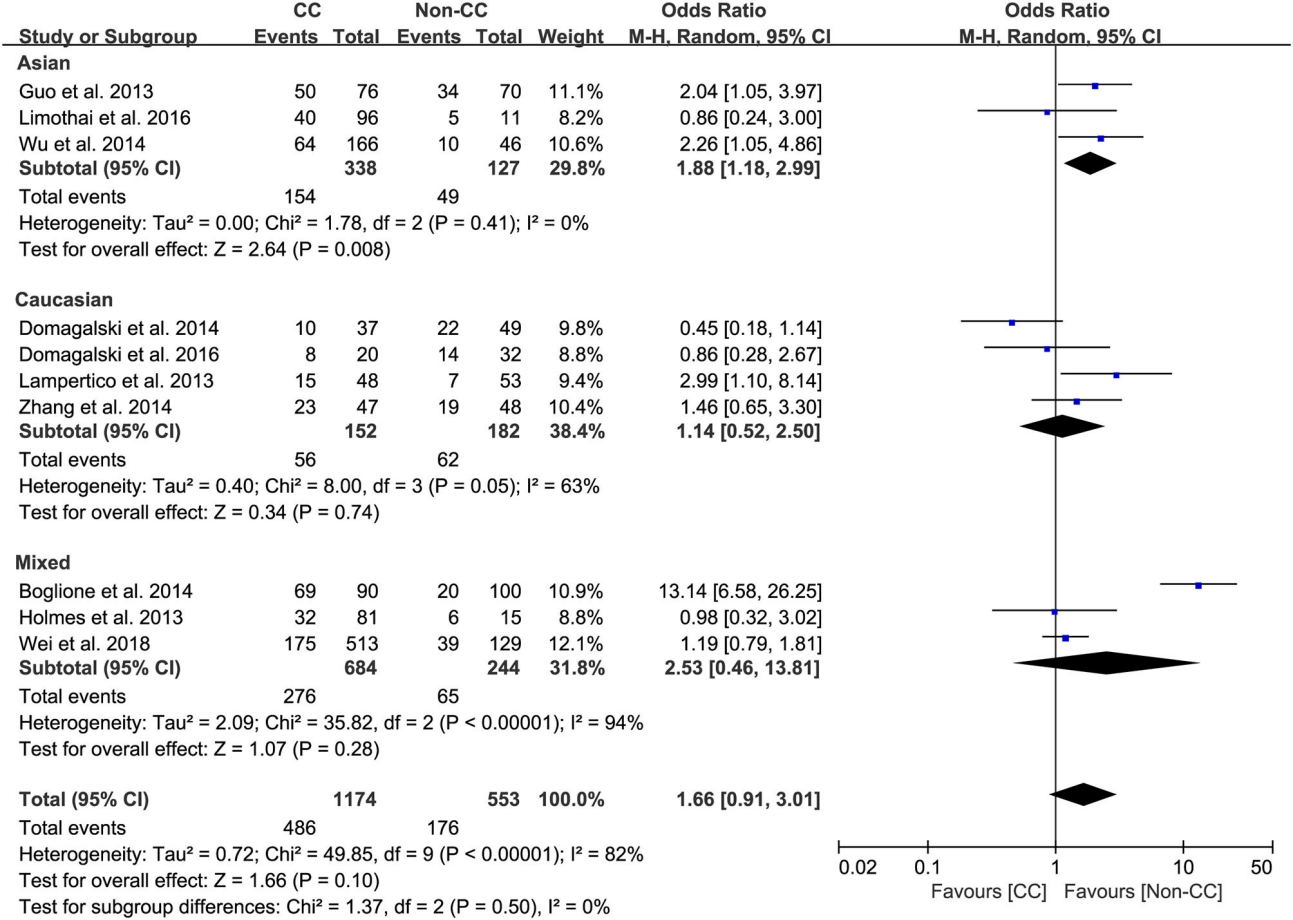
Subgroup analysis of rs12979860, stratified by race.

**Figure 7:**
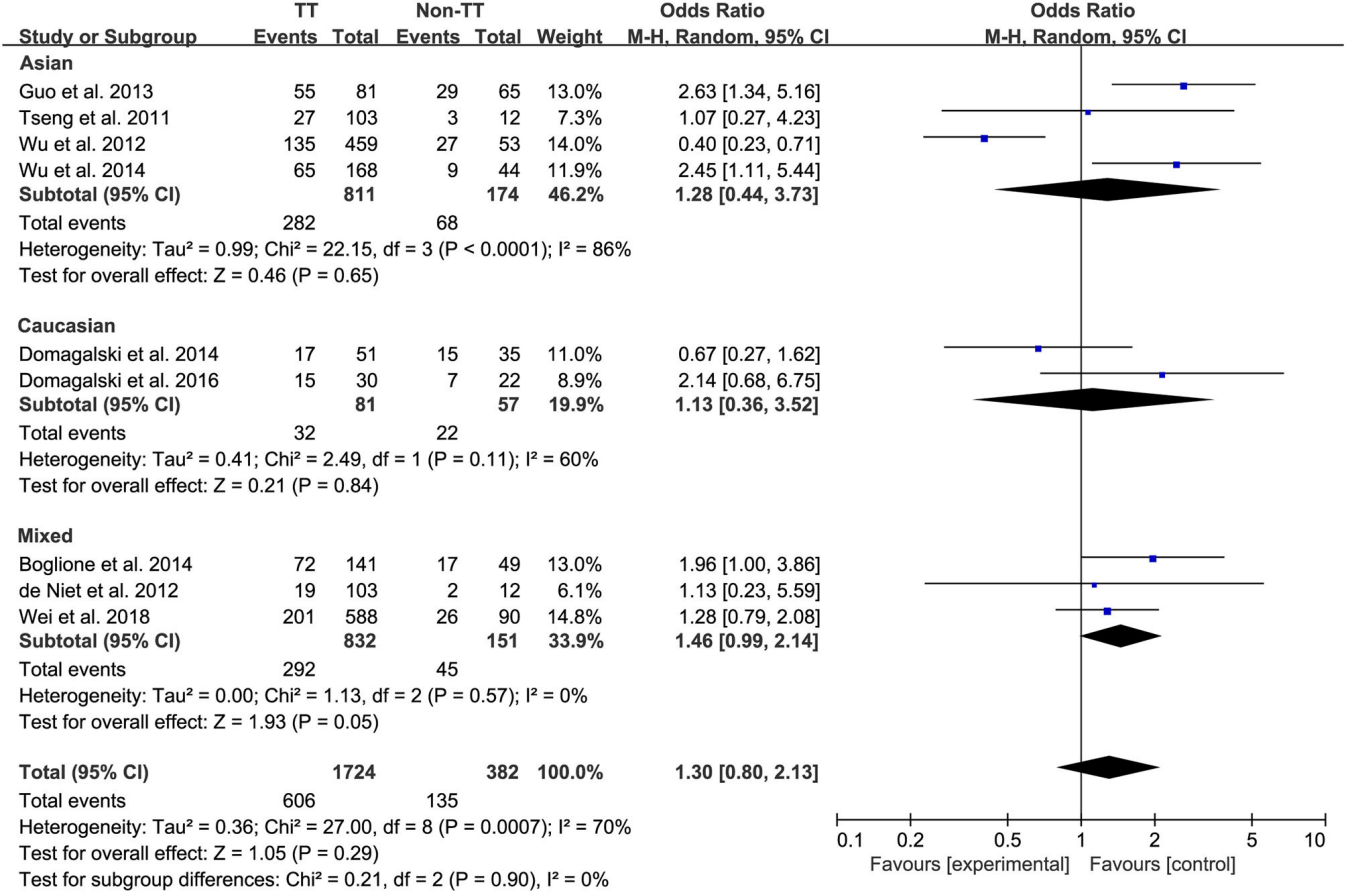
Subgroup analysis of rs12979860, stratified by race.

Among Asian patients with CHB, the *IL-28B rs12979860* CC genotype was associated with a more significant treatment response to PEG-IFN-α (CC vs. non-CC: OR 1.88, 95% CI 1.18–2.99, *I*^2^ = 0%, Figure 6). However, no significant differences were observed among the Caucasian CHB population. In addition, there was no association between the SNP *rs8099917* with the incidence of treatment response among Asian or Caucasian populations with CHB (Figure 7).

## Discussion

To date, there are three identified restriction fragment length polymorphisms (*rs12980275, rs12979860*, and *rs8099917*) of *IL-28B*, which have been studied in relation to the therapeutic efficacy of PEG-IFN-α in patients with CHB. Over the last few years, the association between *IL-28B* polymorphisms and the efficacy of PEG-IFN-α treatment in patients with CHB remains controversial. Many confounding factors can influence the results, such as sample sizes, different genotyping methods, ethnic differences, lifestyle, and environment.

In the present meta-analysis, we evaluated the association between *IL-28B* polymorphisms and treatment response in patients with CHB treated with PEG-IFN. We performed a meta-analysis of the correlation of *IL-28B rs12979860* C/T, *rs12980275* A/G, and *rs8099917* polymorphisms and therapeutic outcomes, including virological response, serological response, biochemical response, or combined response, of PEG- IFN in patients infected with HBV. In this study, we found that there was no significant association between *rs12979860* (CC vs. non-CC), *rs8099917* (TT vs. non-TT) or *rs12980275* (AA vs. non-AA) and treatment response to PEG-IFN-α in all patients with HBV infection. However, in predefined subgroup analysis, we found that among patients with HBeAg-negative CHB, the *rs12979860* CC genotype and *rs8099917* TT genotype were associated with significant treatment response to PEG-IFN-α. Among Asian patients with CHB, the *IL-28B rs12979860* CC was significantly associated with promising treatment response to PEG-IFN-α.

Taken together, the controversy regarding IL-28B polymorphisms and the efficacy of PEG-IFN-α treatment in patients with CHB is especially apparent in the Asian population, as some studies have reported contradictory results (14, 15). Comparing 338 patients with *rs12979860* CC genotype and 127 *rs12979860* non-CC, the results showed that patients with *rs12979860* CC genotype tended to achieve a better treatment response to PEG-IFN-α (OR 1.88, 95% CI 1.18–2.99, *I*^2^ = 0%, Figure 6). No significant differences were observed among the other populations with CHB and *IL-28B* polymorphisms. Thus, *IL-28B rs12979860* CC can act as a predictor for the therapeutic efficacy of PEG-IFN-α in Asian patients with CHB. Previous studies have found that the *IL-28B* polymorphism is an additional predictor of PEG-IFN-α therapy in patients with HBeAg-negative CHB (27). Our results revealed *IL-28B rs12979860* (OR 2.78, 95% CI 1.00–7.76, *I*^2^ = 83%, Figure 4) and *rs8099917* (OR 2.16, 95% CI 1.35–3.48, *I*^2^ = 0%, Figure 5) genotypes could be used to predict curative effects, which is consistent with previous reports.

This study is the first to evaluate the value of three SNPs of *IL-28B* to predict the therapeutic outcomes of PEG-IFN-based treatment in patients with CHB. Nonetheless, our study has some limitations, and the study findings need to be interpreted cautiously. First, since none of the studies included in this meta-analysis were randomized trials, potential bias and confounding factors (e.g., age, sex, baseline HBV DNA, and medication history) could not be well-controlled. Notably, Boglione et al. (19) and Sonneveld et al. (28) performed multivariate analysis, and the adjusted OR indicated that the SNPs were associated with prognostic significance in assessing treatment response in patients with CHB. Boglione et al. (19) found that CC and TT genotypes were related to higher rates of virological response (CC vs. non-CC: adjusted OR 4.29, 95% CI 1.59-11.58; TT vs. non-TT: adjusted OR 3.75, 95% CI 1.24–11.36). Similarly, Sonneveld et al. (28) indicated that more patients with CHB with CC or AA genotype achieved HBeAg seroconversion (CC vs. non-CC: adjusted OR 2.89, 95% CI 1.15–7.80; AA vs. non-AA: adjusted OR 3.16, 95% CI 1.26–8.25). Thus, our results should be further validated in more well-designed trials that fully control potential confounders to rule out alternative explanations.

Another important limitation was the significant heterogeneity observed in the analyses. The existence of clinical heterogeneity is expected to lead to a degree of statistical heterogeneity in the results. The definitions of outcomes were inconsistent among the included studies. Four studies employed the single virological response as the primary outcome, while other studies used the virological response combined with serological or biochemical response as the primary outcome. In addition, the baseline characteristics of patients were inconsistent among included studies as well. Subgroup analysis of studies that only included patients with HBeAg-negative CHB found that patients with CC and/or TT genotype had a high incidence of treatment response to PEG-IFN-α. However, no difference was observed among the populations with positive HBeAg.

## Conclusion

These results indicate that *IL-28B* polymorphism may be related to higher treatment response in patients infected with HBV treated with PEG-IFN. Therefore, detection of the *IL-28B* polymorphism may be an effective biomarker for predicting the treatment response of PEG-IFN-based therapy of patients with CHB. Regarding the limitations mentioned above, further trials warranted to better supplement and confirm our findings.

## Data Availability Statement

The original contributions presented in the study are included in the article/supplementary material, further inquiries can be directed to the corresponding author/s.

## Author Contributions

S-YY, Y-RH, G-SG, K-HL, and ZH provided substantial contribution to the conception, drafting, editing, and final approval of this manuscript. All authors contributed to the article and approved the submitted version.

## Conflict of Interest

The authors declare that the research was conducted in the absence of any commercial or financial relationships that could be construed as a potential conflict of interest.
